# Are very high rates of exogenous carbohydrate ingestion (>90 g/hr) sufficient or indeed necessary to run a sub-2hr marathon? An analysis of the model predictions of Lukasiewicz and colleagues

**DOI:** 10.3389/fnut.2024.1507572

**Published:** 2025-01-08

**Authors:** Timothy D. Noakes, Philip J. Prins

**Affiliations:** ^1^Department of Medical and Wellness Science, Cape Peninsula University of Technology, Cape Town, South Africa; ^2^Department of Exercise Science, Grove City College, Grove City, PA, United States

**Keywords:** carbohydrate oxidation, fat oxidation, muscle glycogen, carbohydrate ingestion, sub-2hr marathon

## Introduction

Lukasiewicz et al. (1) have published a model which predicts that to run a sub-2hr marathon (sub2hrM), prospective male and female runners will need to oxidize ingested (exogenous) carbohydrate (CHO) at respective rates of 93 and 108 g/hr during that race. Their predictions are dependent on the assumptions of their model.

### The ability to run at 21.1 km/hr for 2 h requires high rates of (obligatory) CHO oxidation

Their first assumption is that to run at 21.1 km/hr for 2 h requires a high rate of CHO oxidation, intitially from muscle glycogen. But once muscle glycogen concentrations fall below 32% of the starting value, to prevent any slowing of pace thereafter, essentially all the runners' energy must come from the oxidation of ingested (exogenous) CHO with a small contribution from liver glycogen. Table 1 details their core predictions, in particular rates of exogenous CHO oxidation required to run a sub2hrM. These predictions raise a number of interesting points.

**Table 1:** Modeled rates of total energy expenditure (A); energy from CHO (B, C) and fat oxidation (D); muscle glycogen content at capacity (start of exercise) (E); muscle glycogen use during exercise (F, G); model predicted additional CHO oxidation from blood glucose and exogenous CHO to balance total CHO requirement (H); model predicted CHO contribution from liver glucose disappearance (I); corrected model predicted rates of exogenous CHO contribution to balance total CHO requirement if liver glucose disappearance is 0 g (J); model predicted rates of exogenous CHO oxidation to balance total CHO oxidation (K); model predicted rates of exogenous CHO oxidation required to balance total CHO requirements for runners of different weights (L).

		**Male**	**Female**
A	Total energy expenditure (kJ/min) [Calculated from Calculated caloric cost, kcal – Table 2 in Lukasiewicz et al. (1)]	88.3	75.7
B	Model predicted energy from CHO oxidation (g/min) [Table 2 in Lukasiewicz et al. (1)]	5.1	4.4
C	Model predicted total CHO oxidation (g) during 120 min exercise. (Row B × 120)	612	528
D	Model predicted energy from fat oxidation (g/min) [Table 2 in Lukasiewicz et al. (1)].	0.07	0.06
E	Muscle glycogen (g) at capacity (start of exercise) [Table 3 in Lukasiewicz et al. (1)]	690	499
F	Muscle glycogen (g) remaining if exercise terminates (1) when muscle glycogen concentration falls to 32% or (2) to 20% of the starting concentration	1.221 2. 138 Difference: 83g	1.160 2. 100 Difference: 60g
G	Total muscle glycogen use (g) if exercise terminates (1) at 32% or (2) at 20% of starting muscle glycogen concentration.	1.469 2. 552	1.339 2. 399
H	Model predicted additional CHO requirement from blood glucose and exogenous CHO (g) contribution to balance total CHO requirement according to the authors' model that exercise terminates when muscle glycogen concentration drops (1) below 32% or (2) 20% of the starting concentration (Row C minus Row G)	1.143 2. 60	1.189 2. 129
I	Model predicted CHO contribution from liver glucose (g) disappearance [Figures 4A, B in Lukasiewicz et al. (1)]	68	49
J	Corrected model predicted total exogenous CHO oxidation (g) required to balance total CHO requirement if hepatic glucose disappearance is 0 g (not the values listed in Row I) (Row H plus Row I).	1.211 2. 128	1.238 2. 178
K	Model predicted exogenous CHO (g/hr) required to balance CHO requirement [Figure 6A in Lukasiewicz et al. (1)].	90	106
L	Model predicted rates of exogenous CHO oxidation (g/min) required to balance total CHO requirements for runners of different weights [From Figure 6C in Lukasiewicz et al. (1)].	70 (50 kg) 82 (54 kg) 105 (58 kg) 120 (62 kg)	90 (42 kg) 103 (46 kg) 114 (50 kg) 128 (54 kg)

Rows A–D: Model predicted CHO and fat oxidation rates. Row E: Muscle glycogen content at capacity (start of exercise). Rows F–G: Contribution of muscle glycogen disappearance to total CHO oxidation if fatigue develops at (1) 32% or (2) 20% of starting muscle glycogen concentration. Row H: Model predicted CHO in addition to that from muscle glycogen oxidation required to balance the total required CHO oxidation under two conditions of muscle glycogen disappearance. The (2) in Rows F–H, and J refers to values if fatigue occurs when muscle glycogen is depleted to 20% of the starting concentration. Row I: Predicted CHO contribution from liver glucose disappearance. From Figures 4A, B in Lukasiewicz et al. (1). Row J: Corrected model predicted rates of exogenous CHO oxidation needed to balance total CHO demand if liver glucose disappearance provides 0g to total CHO requirement. Row K: Model predicted rates of exogenous CHO oxidation required to balance CHO requirement [Figure 6A in Lukasiewicz et al. (1)]. Row L: Model predicted exogenous CHO oxidation rates to balance the total CHO requirement for runners of different ages and either sex. From Figure 6C in Lukasiewicz et al. (1).

### Estimations of muscle glycogen use during the first 90–120 min of a sub2hrM attempt in CHO-adapted athletes

Based on the reported range associated with performance decrements, the model predicts that exercise performance deteriorates once the muscle glycogen content falls below 32% of its starting value which, accordingly to the calculated endogenous glycogen stores remaining at the point of slowing, would leave 221 and 160 grams in males and females respectively (Table 1, Row F).

The authors also argue that “*running activates only approximately 68% of the total lower limb muscle (sic);*” thus fatigue develops when glycogen depletion occurs in those 68% of all lower limb muscle fibers.

But is it possible to run at >90% VO_2_max (2) whilst recruiting just 68% of all the quadriceps muscle fibers (as opposed to 68% of all the muscle fibers in the lower limb)? This is relevant because most studies measure exercise-induced changes in muscle glycogen concentrations in that muscle, rather than in all the lower limb muscles. The authors derive their value of 68% from a study (3) of young female physical education students, described as recreational runners with a mean VO_2_max of 49 ml/kg/min. Yet Sale (4) has calculated that 100% of the quadriceps muscle fibers are activated when cycling at >85%VO_2_max [Figure 2; page 99 in Sale (4)]. Perhaps the value of 68% is not realistic for world class male and female athletes competing in the sub2hrM attempt. However, as we show, which ever percentage is chosen, it has little or no influence on the model's predictions.

In the original study (5), on which this model of Lukasiewietz et al. (1) is ultimately based—specifically that there is an obligatory role for CHO oxidation to sustain endurance exercise performance (6)—subjects terminated exercise when their muscle glycogen concentrations were 13%, 10%, and 20% of their starting concentrations following 3 different diets. If subjects in this model terminated exercise with muscle glycogen concentrations reduced to 20% and not 32% of starting concentrations, an additional 83 and 60 g CHO become available for the male and female athletes respectively (Table 1, Row F) leaving a residual 60 g CHO (30 g/hr) in males and 129 g CHO (64.5 g/hr) in females (Lines 2 in Row H, Table 1) to be provided by liver glucose release and exogenous CHO oxidation.

### Estimated CHO contribution from liver glucose disappearance

Panels A and B in the authors' Figure 4 (1) predict that liver glucose disappearance contributes 68 and 49 g CHO to total CHO oxidation in males and females respectively (Table 1, Row I). This is likely overestimated since high rates of CHO ingestion suppress liver glucose disappearance (7–9).

When modified for these two contestable calculations, the predicted rate of exogenous CHO oxidation needed to run a sub2hrM would be 128–211 g CHO/2hr (64–105.5 g CHO/hr) in males and 178–238 CHO/2 hr (80–119 g CHO/hr) in females (Table 1, Row J) compared to rates of 90 and 106 g CHO/hr predicted in the original model (Table 1, Row K). These calculations support the general accuracy of the authors' original conclusions.

### Estimated rates of fat oxidation during exercise at >90% VO_2_max

The authors' second presumption is that during a sub2hrM, the rate of fat oxidation would be 2–3 kJ/min (0.07 and 0.06 g/min for men and women respectively; Row D; Table 1) providing < 3% of the total energy. However, even at exercise intensities >85% VO_2_max, some well-trained but recreational athletes oxidized fat at rates >1.5 g/min (57 kJ/min) (10) potentially supplying 65%−75% of the 76–89 kJ/min required for males and female athletes to run a sub2hrM (Table 1, Row A). Other studies report high rates of fat oxidation even at moderate to high exercise intensities (11).

**Figure 1:**
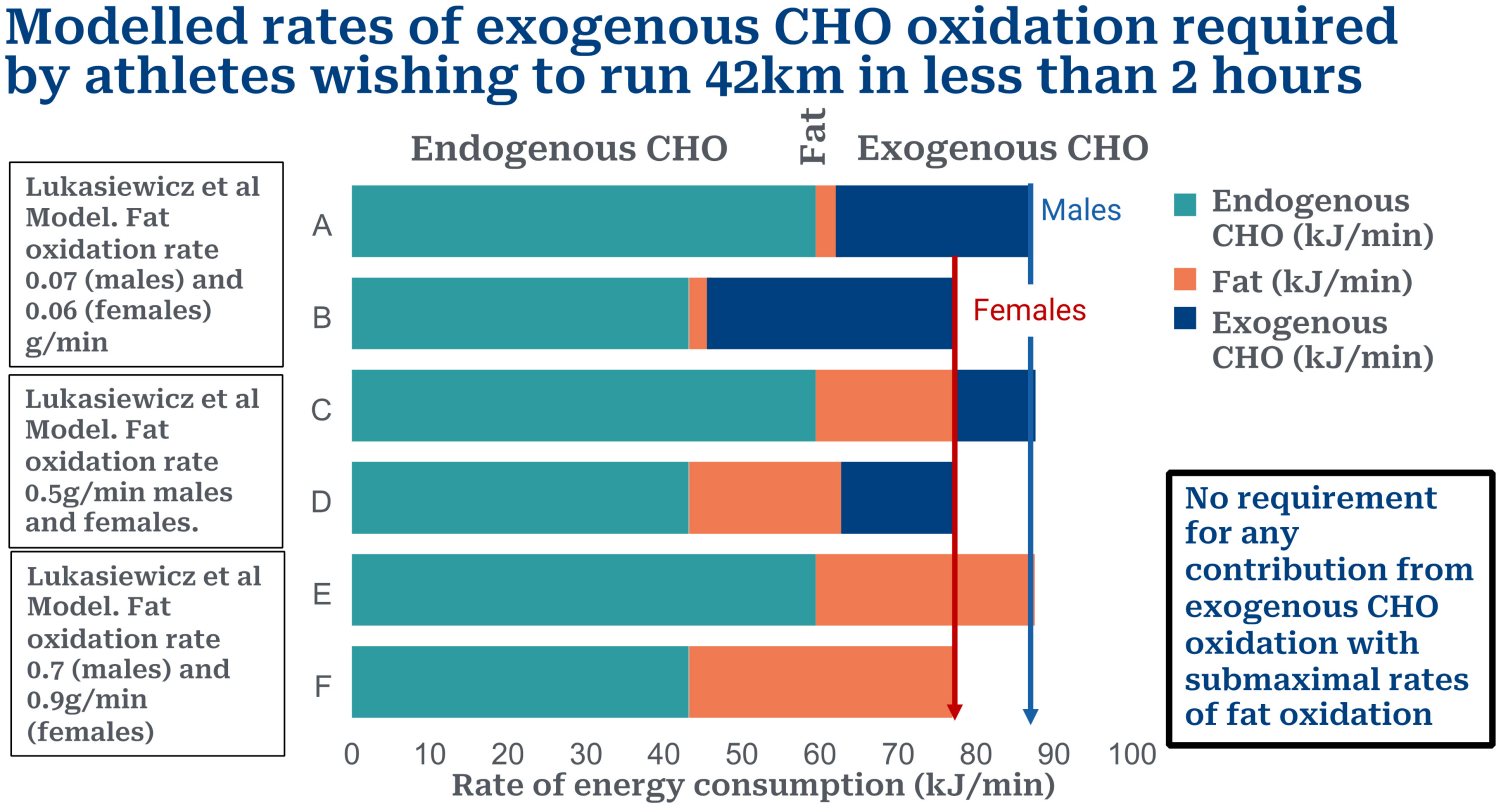
Contributions of endogenous (muscle glycogen), fat and exogenous CHO to total energy expenditure in males and females wishing to run a sub2hrM. Rows A and B according to model of Lukasiewicz et al. (1). Rows C and D when rate of fat oxidation is increased to 0.5 g/min; Rows E and F when rate of fat oxidation is increased to 0.7 g/min for males and 0.9 g/min for males. These higher rates of fat oxidation remove the need for any contribution from exogenous CHO oxidation. Liver glucose disappearance likely makes little or no contribution to energy production at such high rates of CHO ingestion (7–9).

Figure 1 shows the rates of endogenous CHO (muscle glycogen and blood/liver glucose), fat and exogenous CHO oxidation in male (Row A) and female (Row B) athletes running a sub2hrM according to the calculations of Lukasiewicz et al. (1). Increasing the rate of fat oxidation to 0.5 g/min (18 kJ/min) reduces the CHO deficit required from exogenous CHO oxidation to 25 g/hr for males (Row C) and 46 g/min for females (Row D). Increasing the fat oxidation rate to 0.7 g/min in males (Row E) and to 0.9 g/min in females (Row F) removes any requirement for exogenous CHO oxidation to fuel a sub2hrM in either sex.

Thus the model's predictions are critically dependent on the value given to rates of fat oxidation. At present there are few data of rates of fat oxidation in elite athletes exercising at 90% VO_2_max or higher. Furthermore, those data that are available, are usually from studies of athletes habituated to high CHO diets which produce submaximal “peak” rates of fat oxidation (11).

### Rates of total exogenous CHO oxidation calculated from instantaneous rates of exogenous CHO oxidation during exercise

**Figure 2:**
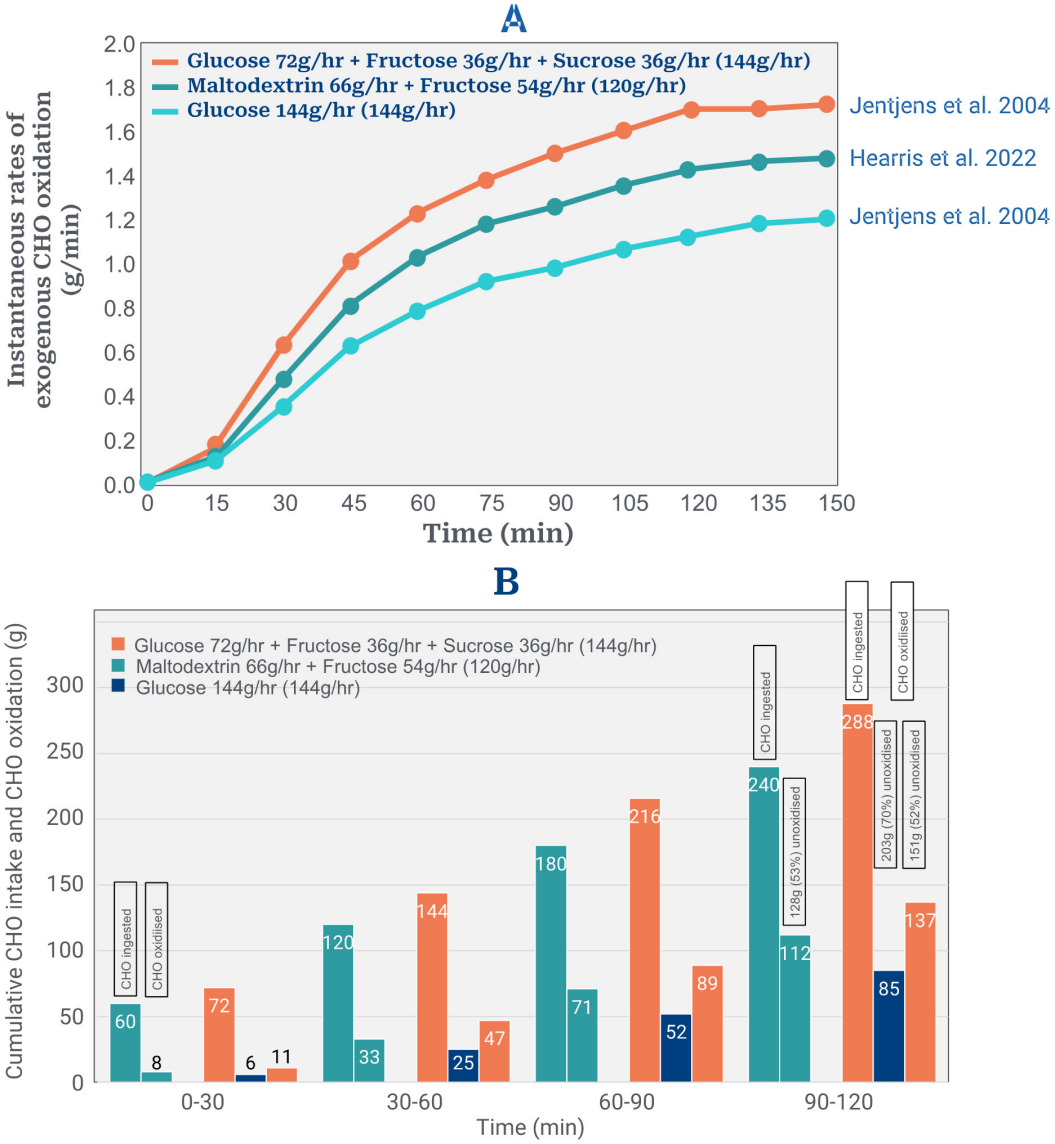
**(A)** Instantaneous rates of exogenous CHO oxidation from three different solutions ingested during 2 h of exercise. Data reproduced from references (12, 13). **(B)** Cumulative amounts of ingested CHO oxidized every 30 min from 3 different solutions. Cumulative amounts of ingested CHO oxidized were estimated from average rates of exogenous CHO oxidation every 30 min using data in **(A)**. Cumulative amounts of unoxidized CHO every 30 min were calculated as amounts ingested minus the average amounts oxidized in that 30 min period.

The third presumption is that ingesting CHO at 90–120 g/min will produce equivalent exogenous CHO rates of 90–120 g/min for the full duration of the sub2hrM. Figure 2 depicts results from the two laboratory studies (12, 13) that have reported the highest rates of exogenous CHO oxidation during exercise. Figure 2A shows instantaneous exogenous CHO oxidation rates in these studies; Figure 2B the cumulative amounts of CHO ingested and oxidized, including the cumulative amounts remaining unoxidized when these experiments concluded.

The study of Jentjens et al. (12) found that the total amount of exogenous CHO oxidized during 2 h of exercise from the optimum solution of glucose, fructose and sucrose was 137 g or 48% of the total ingested amount of 288 g (Figure 2B) leaving 151 g (52%) unoxidized (Figure 2B). Fifty three and 70% of the ingested CHO from the two other tested solutions remained unoxidized after 2 h exercise (Figure 2B) (12, 13).

These data predict that the maximum amount of exogenous CHO oxidation during a sub2hrM in which subjects ingest CHO even at 144 g/hr, would likely be only 137 g (68.5 g/hr). This is substantially less than the rates of 106 g/hr for females and 90 g/hr for males [Figures 6A, B; Lukasiewicz et al. (1)] that the model predicts are required to complete a sub2hrM. Thus the model overpredicts the available total exogenous CHO oxidation amounts by a minimum of 43.6 g/2hr in males and of 75 g/2hr in females.

### Rates of exogenous CHO oxidation fall short of values required to run a sub2hrM

The result is that this model predicts that it is not possible for any athlete to run a sub2hrM relying exclusively on CHO metabolism, even when CHO is ingested at rates of 120–144 g/hr. Rather a reasonable contribution from fat oxidation might make it possible for some athletes to achieve the sub2hrM even whilst ingesting either none or quite small amounts of CHO during the race (Figure 1).

### Is there a time penalty associated with drinking frequently when running at 21.2 km/hr during the sub2hrM attempt?

The fourth assumption is that during the sub2hrM attempt, runners can ingest CHO at very high rates without losing time as a result of frequent drinking. With one exception (14), studies of high rates of CHO ingestion during exercise come from tightly controlled laboratory studies of carbohydrate-adapted cyclists (12, 13, 15–24). The presumption is that results from controlled laboratory experiments of cyclists can be extrapolated to runners competing in an uncontrolled real-world environment, frequently disturbed by the presence of other competitors. For example, intra-abdominal pressures are substantially higher during running than during cycling (25, 26), a difference that will likely influence the ability to replace CHO at high rates when running at >90% VO_2_max for 2 h.

Rowe et al. (14) reported high rates of CHO ingestion (90 g/hr) and total exogenous CHO oxidation (132 g; 66 g/hr) in runners during 2 h of treadmill running but at much lower exercise intensities than are achieved during the sub2hrM. Subjects ingested fluid at a rate of 400 ml/hr from a 22.5% CHO drink and oxidized 73% of the ingested CHO. Cyclists in the study of King et al. (19) achieved similar rates of exogenous CHO oxidation and CHO ingestion (112.5 g/hr) from a much less concentrated CHO solution (11%) but at a much higher fluid ingestion rate (1 L/hr). Hawley et al. (24) reported similarly high rates of CHO ingestion (120 g/hr) by cyclists who ingested a 15% CHO solution at 800 ml/hr. In that study total exogenous CHO oxidation was 45 g/hr, leaving 150 g unoxidized at the end of exercise.

These scientists seem to have discovered that athletes have substantially greater difficulty ingesting fluid at high rates when running than when cycling, even under laboratory conditions. Additionally the movements of the upper body are quite different in running and cycling. The upper body is essentially static during cycling, but rotates substantially when running, increasing with running speed, contributing to increased running efficiency (27). Repeated drinking whilst attempting to ingest CHO at high rates must potentially impairing running performance. Clearly the possible time-wasting effects of frequent drinking during the sub2hrM need to be quantified.

### High rates of CHO ingestion during exercise may have metabolic effects not considered in this model

The final assumption is that ingesting CHO will not produce metabolic effects that could impair running performance. Hawley et al. (24) found that the ingestion of 120 g CHO/hr during exercise at 70% VO_2_max raised blood insulin concentrations, reducing the rate of fat oxidation from ~0.94 to ~0.17 g/min. Total muscle glycogen use during 125 min of exercise was 140 g, 67% greater than in a control group who received enough CHO by infusion to maintain euglycemic blood levels. Similarly the intravenous infusion of 252 g of glucose during 2 h of cycling exercise (126 g/hr) increased total muscle glycogen use by 40 g (28).

## Discussion

The authors have gone to extraordinary lengths to develop a model of the rates of exogenous carbohydrate (CHO) ingestion required to run a sub2hrM. We argue that their model contains two potential flaws that might reverse their conclusions.

First, their model requires that fat oxidation makes no significant contribution to energy use during exercise at >90%VO_2_max. Whilst this is the accepted doctrine for the past century or so, more recent studies find that athletes chronically adapted to low-CHO diets can achieve high rates of fat oxidation even during exercise at >85% VO_2_max (10, 11). We show that rates of fat oxidation of 0.7 g/min in males and 0.9 g/min in females would, according to the predictions of this model, allow athletes of either sex to run a sub2hrM without requiring any additional energy from exogenous CHO oxidation.

Second, the authors assume that high rates of ingested CHO produce equivalent high rates of total exogenous CHO oxidation during exercise. However, studies of high rates of CHO ingestion during exercise show that, at best, only 50% of the ingested CHO is oxidized during the first 2 h of exercise (Figure 2B).

When this latter correction is made to this model, the prediction must be that it will not be possible for a male or female athlete to run a sub2hrM relying purely on CHO metabolism. But with a reasonable contribution from fat oxidation, the sub2hrM might be possible in some athletes even with little or no contribution from the oxidation of exogenous CHO during exercise.

A limitation of this discussion is that it remains theoretical in nature; therefore, further empirical investigation is necessary to validate and substantiate the proposed concepts. Future experimental research should aim to test these hypotheses and explore their practical implications in greater depth. Additionally, it should be acknowledged that no cellular or tissue-level data measurements were obtained from either group, potentially limiting the direct applicability of our findings. Moreover, our calculations did not account for the contributions of amino acids and ketone bodies, which may have influenced the metabolic outcomes considered.

## References

[1] Lukasiewicz CJ Vandiver KJ Albert ED Kirby BS Jacobs RA. Assessing exogenous carbohydrate intake needed to optimize human endurance performance across sex: insights from modeling runners pursuing a sub-2-hour marathon. J Appl Physiol. (2024) 136:158–76. 10.1152/japplphysiol.00521.2023 38059288

[2] Jones AM Kirby BS Clark IE Rice HM Fulkerson E Wylie LJ. Physiological demands of running at 2-hour marathon race pace. J Appl Physiol. (2021) 130:369–79. 10.1152/japplphysiol.00647.2020 33151776

[3] Sloniger MA Cureton KJ Prior BM Evans EM. Lower extremity muscle activation during horizontal and uphill running. J Appl Physiol. (1997) 83:2073–9. 10.1152/jappl.1997.83.6.2073 9390983

[4] Sale DG. Influence of exercise and training on motor unit activation. Exerc Sport Sci Rev. (1987) 15:95–152. 10.1249/00003677-198700150-00008 3297731

[5] Bergström JB Hermansen L Hultman E Saltin B. Diet, muscle glycogen and physical performance. Acta Physiol Scand. (1967) 71:140–50. 10.1111/j.1748-1716.1967.tb03720.x 5584523

[6] Noakes TD. What is the evidence that dietary macronutrient composition influences exercise performance? A narrative review. Nutrients. (2022) 14:862. 10.3390/nu14040862 35215511 PMC8875928

[7] Jeukendrup AE Wagenmakers AJ Stegen JH Gijsen AP Brouns F Saris WH. Carbohydrate ingestion can completely suppress endogenous glucose production during exercise. Am J Physiol Endocrinol Metab. (1999) 276:E672–83. 10.1152/ajpendo.1999.276.4.E672 10198303

[8] Gonzalez JT Fuchs CJ Smith FE Thelwall PE Taylor R Stevenson EJ. Ingestion of glucose or sucrose prevents liver but not muscle glycogen depletion during prolonged endurance-type exercise in trained cyclists. Am J Physiol Endocrinol Metab. (2015) 309:E1032–9. 10.1152/ajpendo.00376.2015 26487008

[9] Bosch AN Dennis SC Noakes TD. Influence of carbohydrate ingestion on fuel substrate turnover and oxidation during prolonged exercise. J Appl Physiol. (1994) 76:2364–72. 10.1152/jappl.1994.76.6.2364 7928859

[10] Prins PJ Noakes TD Buga A D'Agostino DP Volek JS Buxton JD. Low and high carbohydrate isocaloric diets on performance, fat oxidation, glucose and cardiometabolic health in middle age males. Front Nutr. (2023) 10:1084021. 10.3389/fnut.2023.1084021 36845048 PMC9946985

[11] Noakes TD Prins P Volek JS D'Agostino DP Koutnik AP. Low carbohydrate high fat ketogenic diets on the exercise crossover point and glucose homeostasis. Front Physiol. (2023) 14:1150265. 10.3389/fphys.2023.1150265 37057184 PMC10086139

[12] Jentjens R Achten J Jeukendrup AE. High oxidation rates from combined carbohydrates ingested during exercise. Med Sci Sports Exerc. (2004) 36:1551–8. 10.1249/01.MSS.0000139796.07843.1D 15354037

[13] Hearris MA Pugh JN Langan-Evans C Mann SJ Burke L Stellingwerff T. 13C-glucose-fructose labeling reveals comparable exogenous CHO oxidation during exercise when consuming 120 g/h in fluid, gel, jelly chew, or coingestion. J Appl Physiol. (2022) 132:1394–406. 10.1152/japplphysiol.00091.2022 35446596

[14] Rowe JT King R King AJ Morrison DJ Preston T Wilson OJ. Glucose and fructose hydrogel enhances running performance, exogenous carbohydrate oxidation, and gastrointestinal tolerance. Med Sci Sports Exerc. (2022) 54:129–40. 10.1249/MSS.0000000000002764 34334720

[15] King AJ O'Hara JP Arjomandkhah NC Rowe J Morrison DJ Preston T. Liver and muscle glycogen oxidation and performance with dose variation of glucose–fructose ingestion during prolonged (3 h) exercise. Eur J Appl Physiol. (2019) 119:1157–69. 10.1007/s00421-019-04106-9 30840136 PMC6469629

[16] Jeukendrup AE Moseley L Mainwaring GI Samuels S Perry S Mann CH. Exogenous carbohydrate oxidation during ultraendurance exercise. J Appl Physiol. (2006) 100:1134–41. 10.1152/japplphysiol.00981.2004 16322366

[17] Jentjens RL Moseley L Waring RH Harding LK Jeukendrup AE. Oxidation of combined ingestion of glucose and fructose during exercise. J Appl Physiol. (2004) 96:1277–84. 10.1152/japplphysiol.00974.2003 14657042

[18] Podlogar T Bokal Š Cirnski S Wallis GA. Increased exogenous but unaltered endogenous carbohydrate oxidation with combined fructose-maltodextrin ingested at 120 g h– 1 versus 90 g h– 1 at different ratios. Eur J Appl Physiol. (2022) 122:2393–401. 10.1007/s00421-022-05019-w 35951130 PMC9560939

[19] King AJ O'Hara JP Morrison DJ Preston T King RF. Carbohydrate dose influences liver and muscle glycogen oxidation and performance during prolonged exercise. Physiol Rep. (2018) 6:e13555. 10.14814/phy2.13555 29333721 PMC5789655

[20] Baur DA Schroer AB Luden ND Womack CJ Smyth SA Saunders MJ. Glucose-fructose enhances performance versus isocaloric, but not moderate, glucose. Med Sci Sports Exerc. (2014) 46:1778–86. 10.1249/MSS.0000000000000284 25134001

[21] Wallis GA Rowlands DS Shaw C Jentjens R Jeukendrup AE. Oxidation of combined ingestion of maltodextrins and fructose during exercise. Med Sci Sports Exerc. (2005) 37:426–32. 10.1249/01.MSS.0000155399.23358.82 15741841

[22] O'Brien WJ Rowlands DS. Fructose-maltodextrin ratio in a carbohydrate-electrolyte solution differentially affects exogenous carbohydrate oxidation rate, gut comfort, and performance. Am J Physiol Gastrointest Liver Physiol. (2011) 300:G181–9. 10.1152/ajpgi.00419.2010 21071509

[23] Trommelen J Fuchs CJ Beelen M Lenaerts K Jeukendrup AE Cermak NM. Fructose and sucrose intake increase exogenous carbohydrate oxidation during exercise. Nutrients. (2017) 9:167. 10.3390/nu9020167 28230742 PMC5331598

[24] Hawley JA Bosch AN Weltan SM Dennis SC Noakes TD. Effects of glucose ingestion or glucose infusion on fuel substrate kinetics during prolonged exercise. Eur J Appl Physiol Occup Physiol. (1994) 68:381–9. 10.1007/BF00843733 8076616

[25] Shaw JM Hamad NM Coleman TJ Egger MJ Hsu Y Hitchcock R. Intra-abdominal pressures during activity in women using an intra-vaginal pressure transducer. J Sports Sci. (2014) 32:1176–85. 10.1080/02640414.2014.889845 24575741 PMC3992988

[26] Dietze-Hermosa M Hitchcock R Nygaard IE Shaw JM. Intra-abdominal pressure and pelvic floor health: should we be thinking about this relationship differently? Urogynecology. (2020) 26:409–14. 10.1097/SPV.0000000000000799 32574030 PMC8974352

[27] Lang C Schleichardt A Warschun F Walter N Fleckenstein D Berkel F. Relationship between longitudinal upper body rotation and energy cost of running in junior elite long-distance runners. Sports. (2023) 11:204. 10.3390/sports11100204 37888531 PMC10611096

[28] Hawley JA Bosch AN Weltan SM Dennis SC Noakes TD. Glucose kinetics during prolonged exercise in euglycaemic and hyperglycaemic subjects. Pflugers Arch. (1994) 426:378–86. 10.1007/BF00388300 8015888

